# Feasibility of hypnosis for analgosedation during atrial fibrillation ablation, including pulsed field and radiofrequency techniques: a pilot study

**DOI:** 10.1093/europace/euag087

**Published:** 2026-05-14

**Authors:** Marco Scaglione, Enrico Guido Spinoni, Alberto Battaglia, Nicolò Pellegrini, Marco Gagliardi, Natascia Cerrato, Francesco Geuna, Andrea Lamanna, Milena Muro, Gabriella Amerio, Paula Fernandes, Alessandro Bianchi, Enrico Facco, Domenico Caponi

**Affiliations:** Electrophysiology Laboratory, Cardinal Massaia Hospital, Asti, Italy; Electrophysiology Laboratory, Cardinal Massaia Hospital, Asti, Italy; Electrophysiology Laboratory, Cardinal Massaia Hospital, Asti, Italy; Electrophysiology Laboratory, Cardinal Massaia Hospital, Asti, Italy; Electrophysiology Laboratory, Cardinal Massaia Hospital, Asti, Italy; Electrophysiology Laboratory, Cardinal Massaia Hospital, Asti, Italy; Electrophysiology Laboratory, Cardinal Massaia Hospital, Asti, Italy; Electrophysiology Laboratory, Cardinal Massaia Hospital, Asti, Italy; Electrophysiology Laboratory, Cardinal Massaia Hospital, Asti, Italy; Electrophysiology Laboratory, Cardinal Massaia Hospital, Asti, Italy; Electrophysiology Laboratory, Cardinal Massaia Hospital, Asti, Italy; Division of Anesthesiology, Cardinal Massaia Hospital, Asti, Italy; Department of Neurosciences, University of Padua, Padua, Italy; Electrophysiology Laboratory, Cardinal Massaia Hospital, Asti, Italy

**Keywords:** Pulsed field ablation, Sedation protocol, Atrial fibrillation, Hypnosis

## Introduction

Atrial fibrillation (AF) ablation typically requires general anaesthesia (GA) or deep sedation (DS).^1–3^ GA is often preferred for pulsed field ablation (PFA) to manage pain and muscular/diaphragmatic contractions.^4,5^ Hypnosis has emerged as an alternative technique for managing procedural pain and anxiety, but it has never been tested during PFA.^6,7^

This prospective non-randomized pilot study assesses the feasibility of hypnosis as analgosedation strategy, with DS/GA as bailout, across three ablation modalities: PFA, very high-power short duration (vHPSD) and temperature-controlled radiofrequency (RF) delivery.

## Materials and methods

This prospective pilot study described an exploratory feasibility experience across three different ablation technologies in consecutive paroxysmal or persistent AF patients:

- Cohort 1: PFA using a pentaspline catheter (Farapulse™, Boston Scientific).
- Cohort 2: Temperature-controlled RF ablation via ThermoCool SmartTouch™ catheter (Biosense Webster).
- Cohort 3: vHPSD RF ablation (90W, 4 s) using a QDOT Micro™ catheter (Biosense Webster).

Hypnosis was authorized by internal ethical committee and embedded in the informed consent. The protocol adhered to the Declaration of Helsinki.

Primary study endpoint was the feasibility of a predefined workflow in AF ablation using hypnosis, having DS or GA as a bailout strategy. Escalation criteria to DS/GA were: not tolerated pain occurrence during the procedure, uncontrolled movements, and/or haemodynamic instability.

Secondary endpoints included analgesic dosage, patient-perceived pain, procedural discomfort manageability, and total duration.

## Hypnosis and sedation protocols

**Figure 1:**
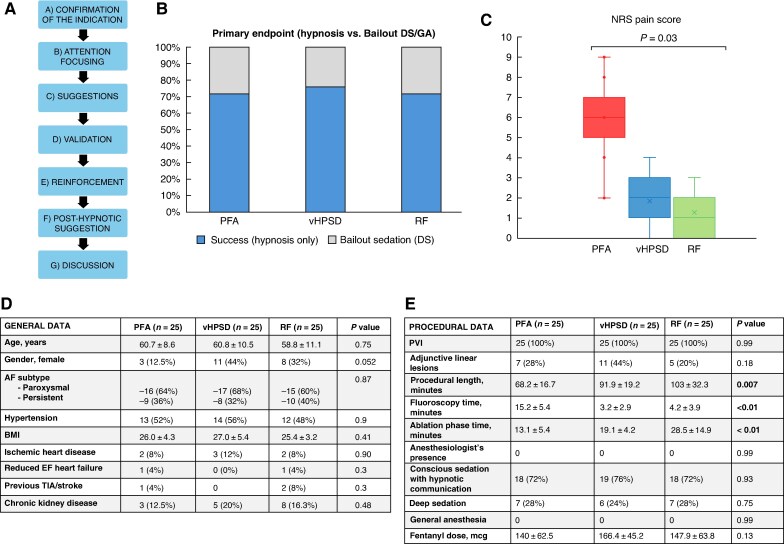
Panel *A*. Procedural workflow of hypnotic communication during atrial fibrillation procedure.^6^ Panel *B*. Primary endpoint: Percentage of patients in the study groups completing the procedure with hypnosis or with deep sedation (DS) or general anaesthesia (GA) as bailout. Panel *C*. Comparison of pain score assessment by Number Rating Scale (NRS) Score in the study groups. Panel *D*. General characteristics of the study groups. Panel *E*. Index procedural data in the study groups. AF = atrial fibrillation; BMI = body mass index; EF = ejection fraction; PFA = pulsed field ablation; PVI = pulmonary vein isolation; RF = radiofrequency; TIA = transient ischaemic attack; vHPSD = very high power short duration.

No assessment of hypnotic susceptibility or standardized scale was performed, avoiding any selection bias. A preliminary talk was used to evaluate contraindications (cognitive impairment or psychiatric disorders), linguistic barrier, and inability to comply with suggestion orders during the preliminary interview. In such cases, hypnosis was not proposed, while applied in all the remaining patients, administered in the EP lab by trained healthcare professionals following a validated workflow (*Figure 1*, Panel *A*).^6^ Patients remained in hypnotic state throughout the procedure and were dehypnotized upon completion. Hypnosis served as an adjunct to local anaesthesia (lidocaine 2%) and systemic analgesia (fentanyl 1–2 mcg/kg and paracetamol 1000 mg). If required, bailout DS (ketamine 1.5–2 mg/kg, or dexmedetomidine 1 mcg/kg/h) was managed by EP personnel, with an anaesthesiologist available on-call for GA.^2,3^

## Pain assessment and statistical analysis

The day after a follow-up questionnaire was administered testing:

- procedural pain and manageability using Number Rating Scale (NRS, 0–10),
- tolerability and willingness to undergo future procedures under hypnosis.

Continuous variables are presented as mean ± SD, and categorical variables as frequencies (%). Analysis was performed using Stata Release 18 (StataCorp LLC).

## Results

Seventy-five consecutive patients were included (*n* = 25 PFA patients, *n* = 25 vHSPD patients, *n* = 25 RF patients). Population characteristics are listed in *Figure 1*, Panel *D*. Female gender was predominant in vHSPD patients (*P* = 0.053).

Procedural data are listed in *Figure 1*, Panel *E*. Preliminary talk excluded 20 patients (26.7%) for hypnosis due to contraindications or inability to comply with suggestion orders, who underwent DS. No cases required GA. All the patients whom hypnosis was offered accepted the proposed strategy.

The ablation was effectively carried out with hypnosis in 18 (72%) PFA patients, 19 (76%) vHPSD patients, and 18 (72%) RF patients (*Figure 1*, Panel *B*). Remaining cases underwent DS (*n* = 20, 26.7%). None of the patients under hypnosis claimed not tolerated pain during the procedure.

Perceived pain score was higher in PFA patients (*P* = 0.03) (*Figure 1*, Panel *C*), but was managed by the patient without escalation to DS/GA in any case.

Manageability and patients’ tolerance was not significantly different (PFA 8.5 ± 1.3, vHPSD 8.6 ± 1.2, RF 8.8 ± 1.1, *P* = 0.75). Accordingly, 23 (92%) in the PFA, 24 (96%) in the vHPSD, and 23 (92%) in the RF group answered positively to the questionnaire.

Fentanyl doses were similar in the study groups (*P* = 0.13) who underwent hypnosis.

Procedural duration was 83.3 ± 24.7 min with hypnosis and 98.7 ± 28.4 min with DS.

Neither sedation nor procedure-related adverse events were recorded.

## Discussion

Main results of our non-randomized pilot study are:

- The ablation procedure was effectively carried out with hypnosis in a high rate of PFA (72%), vHPSD (76%), and RF (72%) patients and no need for escalation emerged.
- Ablation with PFA was associated with stronger intraoperative pain; however with optimal tolerability and perceived comfort.
- A selection for candidates to hypnosis was applied through a preliminary talk and 20 out of 75 patients for hypnosis were excluded due to contraindications or poor compliance.

The high tolerability of PFA under hypnosis, despite reported sensory intensity, aligns with the International Association for the Study of Pain definition of pain as a complex experiential construct rather than mere nociception, entailing a complex, dynamic interplay between physical and psychological factors and environment.^8^ Hypnosis can decouple the sensory dimension from affective-motivational distress modulating the pain neuromatrix.^9,10^

In this study, targeted suggestions reframed intraoperative sensations as transient and benign, resulting in high patient satisfaction; notably, 92% of participants expressed a willingness to repeat the procedure. These exploratory findings support the hypothesis of potential feasibility of hypnosis in PFA, which might reduce reliance on systemic analgesics (DS or GA). Therefore, hypnosis may enhance procedural efficiency as the rapid induction and patient relaxation facilitates a stable operative environment.

Finally, no procedural related adverse events were seen in all cases.

Anyway, due to the small size of the population, extended comparative data are required to confirm the safety profile of the procedure.

To our knowledge, the present study is the first report investigating the potential feasibility of hypnosis during PFA of AF.

## Limitations

This is a limited sample size exploratory non-randomized pilot study. Female gender was underrepresented. Only one single-shot pentaspline PFA catheter was included. This study protocol might not be applicable in other centres. Hypnosis is operator and patient dependent, requiring trained professionals. Furthermore, no formal assessment of hypnotic susceptibility or standardized scale was applied. Noteworthy, approximately 26.7% were excluded from hypnosis based on predefined criteria, possibly resulting in a selection bias. Lastly, the criteria for escalation to DS/GA remain partly subjective and operator-dependent.

## Conclusions

An approach implying the use of hypnosis as an adjunctive technique for analgo-sedation appears feasible during PFA with a pentaspline catheter for AF, when appropriately selected by experienced physicians.

## Data Availability

Data available upon reasonable request.
